# Angular-Spectral Characteristics of Acousto-Optic Tunable Filters Based on Mercurous Halide Crystals

**DOI:** 10.3390/ma17050967

**Published:** 2024-02-20

**Authors:** Huijie Zhao, Chi Cheng, Qi Guo, Kai Yu, Yutian Yang

**Affiliations:** 1 School of Instrumentation and Opto-Electronic Engineering, Beihang University, Beijing 100191, China; chengchi2015@buaa.edu.cn (C.C.); yukai@buaa.edu.cn (K.Y.); sweety0651@buaa.edu.cn (Y.Y.); 2 Institute of Artificial Intelligence, Beihang University, Beijing 100191, China; 3 Aerospace Optical-Microwave Integrated Precision Intelligent Sensing, Key Laboratory of Ministry of Industry and Information Technology, Beihang University, Beijing 100191, China; 4 Qingdao Research Institute of Beihang University, Qingdao 266104, China

**Keywords:** mercurous halides, AOTF, phase matching, separation angle

## Abstract

The angular and spectral properties crucial for the functionality of acousto-optic (AO) devices are determined by phase-matching geometries in AO interactions. In applications such as spectral imagers based on acousto-optic tunable filters (AOTFs), systematic throughput is constrained by the angle separating diffracted and transmitted light. This research introduces an analytical model that elucidates the angular-spectral properties of diffracted beams in mercurous halide crystals. These crystals possess a wide transmissive spectral range, from visible light to long-wave infrared light. The study computes and confirms correlations between the separation angle and parameters including incident angle, polarization, acoustic angle, and crystal birefringence. Experimental validation conducted on mercurous halide and tellurium dioxide crystals demonstrates that higher birefringence in crystals significantly amplifies the separation angle, augmenting the device’s performance. The study contributes to the development of devices with large separation angles during the design phase, enhancing systematic throughput in spectral imaging applications.

## 1. Introduction

When an acoustic wave travels through an optically transparent medium, it induces a periodic modulation of the refractive index, a phenomenon referred to as the elasto-optical effect. This effect causes incident light to diffract in one or multiple directions and is extensively employed in acousto-optic (AO) devices for modulating light across multiple parameters [1]. One notable such device is the acousto-optic tunable filter (AOTF), a compact, electronically addressable, solid-state tunable filter that has been extensively studied for use in space exploration [2], spectral microscopy [3], color reproduction [4], laser control [5], and spectral imaging [6]. AOTFs operate with an AO crystal, wherein incident light undergoes diffraction due to acoustic waves generated by ultrasonic transducers. AOTF-based spectral imaging systems, known for their rapid tuning, reliability, repeatability, and flexible spectral channel switching, enable quicker acquisition of spectral data in scenes compared to conventional push-broom systems [7].

Spectral imaging systems require a wide angular aperture to optimize energy collection, which in turn improves signal quality and enables the capture of larger imaging frames. Compared to a collinear configuration, the diffracted and transmitted light of non-collinear AOTFs can be spatially separated, making it more convenient to utilize the diffracted light [8]. However, the performance of non-collinear acousto-optic tunable filters has been limited by an extremely small angular aperture (around 1 mrad) due to its sensitivity to incident angles in prior studies [9], restricting their application in imaging. To overcome this challenge, the parallel tangents principle (PTP) was introduced as a fundamental design concept for non-collinear AOTFs, with the aim of achieving a broader angular aperture [8]. When the relationship between the acoustic angle and incident angle satisfies the PTP, AO diffraction becomes less susceptible to the angle of light incidence. The theoretical formula for the angular aperture of non-collinear AOTFs, proposed by Chang [10], establishes the groundwork for subsequent research on AOTFs and system design, ranging from visible lights to long waves [11], from traditional crystal [12] to novel crystals [13], and from single-channel [14] to multi-channel types [15].

In the AOTF imaging system, the angular aperture is constrained by two factors. Firstly, the diffraction efficiency decreases when monochromatic incidence is inclined [10]. However, when white light is incident, the light outside the angular aperture is also diffracted, with only the central wavelength of diffracted light drifting. This drift can be calibrated both experimentally and theoretically [16]. A proposal has been made to enhance the throughput of AOTF-based imagers by utilizing a super-angular aperture scheme for AOTF spectral imaging systems [17]. As a result, the reception angle of AOTFs in imaging applications has been further expanded through optical design and an analysis of the spectral characterization of AOTF diffraction.

Another limitation of the angular aperture is the separation angle of the AOTF, which is the angle between the diffracted light and the transmitted light. When the reception angle of the incident beam exceeds the separation angle, the images in the zeroth and first diffraction orders overlap spatially [18]. In theory, the use of polarizers in the outgoing optical path can eliminate transmitted light, given that the polarization states of the diffracted light and the incident light are orthogonal in anisotropic AO diffraction. Nevertheless, because of the restricted extinction ratio of the polarizer and the high intensity of transmitted light in comparison to diffracted light, it is not possible to completely filter out the transmitted light, which leads to additional noise. In the other orthogonal direction, however, there is no constraint due to the separation angle [17]. Consequently, the current restrictions on the separation angle of AOTFs remain the primary factor limiting the angular aperture and ultimately the throughput of an AOTF-based imaging system [19].

The development of acoustic-optic technology is inseparable from advancements in material applications, such as the all-optical identification of acoustic waves by noble metal nanoparticles [20]. For the AOTF devices discussed in this article, there is generally a greater focus on high-performance solid-state materials. Mercurous halide acousto-optic crystals, as a novel category of acousto-optic crystals, have proven to be outstanding for crafting broadband spectral elements due to their characteristic features, such as high birefringence [13], a substantial acousto-optic figure of merit that characterizes the diffraction efficiency of acousto-optic devices [21], and a broad transparent range [22]. Nevertheless, their spectral attributes deviate from those of tellurium dioxide (TeO_2_) materials, rendering the design methodologies employed for tellurium dioxide crystal AOTFs unsuitable for the development of mercurous halide (Hg_2_X_2_) AOTF devices. This study explores phase-matching geometries and investigates the impact of the acoustic wavevector, incident wavevector, and optical anisotropy on the separation angle, providing guidance for the design of AOTF devices with large separation angles. The angular properties of light beams diffracted by ultrasound in mercurous halide crystals have been investigated both theoretically and experimentally, and this work includes comparisons of these properties with those of tellurium dioxide.

## 2. Methods

**Figure 1:**
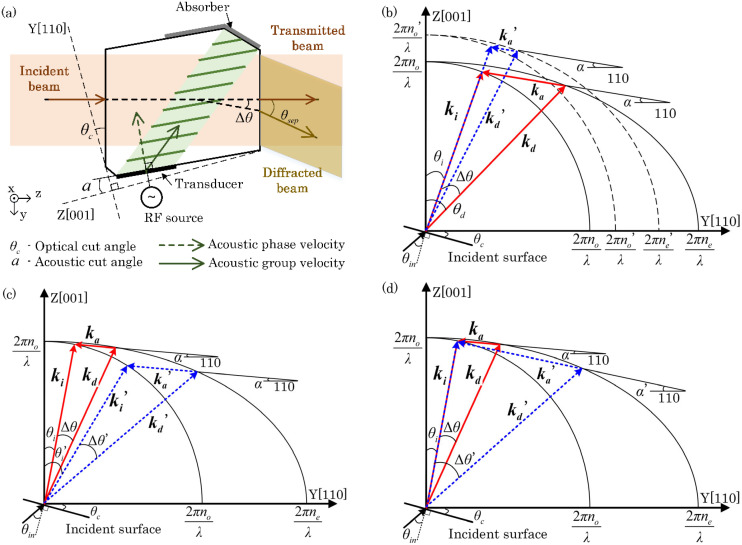
(**a**) Schematic diagram of non-collinear AOTFs. (**b**) Wavevector diagram for crystals with different anisotropies. Dashed lines represent the wavevector curves of the tellurium dioxide crystals and the phase-matching relationships inside the crystals, while solid lines represent the wavevector curves of the mercurous halide crystals, which have larger birefringence, and the phase-matching relationships inside the crystals. (**c**) Wavevector diagram for crystals with different incident angles *θ_i_*. The two sets of phase matching have the same acoustic angle and different incident angles. (**d**) Wavevector diagram for crystals with different acoustic angles *α*. The two sets of phase matching have different acoustic angles and the same incident angle.

The acousto-optic interaction in a uniaxial crystal is depicted in a wavevector diagram, as shown in Figure 1. The orientation and velocity at which the acoustic field travels inside the AOTF device are determined by the crystal’s acoustic cut angle α and the crystal’s acoustic velocity along the crystal axis. This α is the angle between the transducer and the [001] axis. The acousto-optic crystal’s incident surface is cut at a specific angle *θ_c_* to ensure that the incident light beam propagates through the acousto-optic crystal at the desired angle. This optical cut angle *θ_c_* is the angle between the incident surface and the [110] axis.

As the acoustic field of a distinct frequency progresses within the crystal along a specific path, it establishes an acousto-optic grating, resulting in anisotropic Bragg diffraction of incident light. This diffraction diverts light of a specific wavelength to a particular direction, as illustrated in Figure 1a. The anisotropic Bragg diffraction can be described by the phase matching of incident light **k_i_**, acoustic field **k_a_**, and diffracted light **k_d_**. This geometric relationship is described by [1], as follows:

(1) $$k_{i}\pm k_{a}=k_{d}$$

Here, *k_a_* = 2π*f_a_*/*v_a_* and represents the magnitude of the acoustic wavevector, while *k_i_* = 2π*n_i_*/*λ* and *k_d_* = 2π*n_d_*/*λ* and correspond to the wavevectors for the incident and diffracted light, respectively, where *n_i_*, *n_d_* are the refractive indices for the incident and diffracted light. *λ* is the wavelength of diffracted light. *f_a_* is the frequency of acoustic waves generated by a piezoelectric transducer. *v_a_* is the phase velocity of the acoustic wave. The incident angle *θ_i_* and diffraction angle *θ_d_* are defined in the crystallographic coordinate with respect to the crystal axis [001]. The phase-matching relationships among the three vectors can be clearly described using wavevector diagrams, as shown in Figure 1b–d. In this article, only the geometric relationship of acousto-optic phase matching is considered, and variations in intensity are not taken into account. Therefore, losses and their associated effects [23] have not been included in the analysis. Suppose the incidence is ordinarily polarized (o-light) and the end of the incident light’s wavevector **k_i_** falls on the wavevector circle *Y_i_*^2^ + *Z_i_*^2^ = (2πn_o_/*λ*)^2^. For the diffracted light (e-light), the end of the diffracted light’s wavevector falls on the wavevector ellipse *Y_d_*^2^/n_e_^2^ + *Z_d_*^2^/n_o_^2^ = (2π/*λ*)^2^, where n_o_, n_e_ are the principle refractive indices of ordinary and extraordinary light, respectively. The matching constrains the acoustic wavevector to form a closed triangle with the incident and diffracted light wavevectors. The acoustic vector is along the direction of the acoustic phase velocity, with the amplitude determined by frequency *f_a_* and velocity *v_a_*. The velocity of acoustic wave varies with the acoustic cut angle α, given by *v_a_* = (V_110_cos2α + V_001_sin2α)^1/2^ [13], where V_110_ and V_001_ are velocities of the shear slow wave along the [110] and [001] crystal axis.

When the incident light is extraordinarily polarized, *n_i_* varies with incident angle *θ_i_*, given by n_i_ = n_e_’(*θ_i_*) = [(n_o_n_e_)^2^/(n_o_^2^sin^2^*θ_i_* + n_e_^2^cos^2^*θ_i_*)]^1/2^ [13].

Given that the phase-matching condition is satisfied, monochromatic diffracted light is diffracted in a specific direction. The angle between the direction of the diffracted light and the incident light is defined as the separation angle, which consists of the separation angle inside the crystal Δ*θ* and the separation angle outside the crystal *θ_sep_*. Here, *θ_sep_* can be converted from Δ*θ* by using the refraction law at the exit surface of the AOTF device. For the tellurium dioxide and mercurous bromide/chloride crystals, which have relatively high refractive indices (near 2), as shown in Table 1, the separation angle outside the crystal *θ_sep_* can be approximately considered to be around twice the separation angle inside the crystal Δ*θ* when the incident surface and the exit surface of the crystal are parallel to each other. According to simple trigonometric geometric relationships, the separation angle inside the crystal Δ*θ* is derived as follows [18]:

(2) $$\Delta \theta =\arccos[\frac{n_{i}}{n_{d}}\cos(\theta_{i}-\alpha )]-\theta_{i}+\alpha$$

The separation angle inside the crystal Δ*θ* is determined by the ratio of the refractive indices of the incident and diffracted light, as well as the incident angle and the acoustic cut angle, as demonstrated with wavevector diagrams in Figure 1a, Figure 1b and Figure 1c, respectively.

When the wavevectors in the phase-matching relationships have the same incident angle and acoustic cut angle, the birefringence of the acousto-optic crystal alone determines the separation angle inside the crystal. In Figure 1b, the dashed lines represent the wavevector curves of the TeO_2_ crystals and the phase-matching relationships inside the crystals, while the solid lines represent the wavevector curves of the Hg_2_X_2_ crystals, which exhibit larger birefringence, and the phase-matching relationships inside the crystals. The endpoints of the acoustic wavevectors **k_a_** and **k_a_’** lie on the ordinary wavevector circle and the extraordinary wavevector ellipse, respectively. The refractive index of the ordinary light in Hg_2_X_2_ crystals is lower than that in TeO_2_, while the refractive index of the extraordinary light is higher than that in TeO_2_. This result implies that the birefringence in the Hg_2_X_2_ crystal is higher and thus that the in-crystal separation angle Δ*θ* formed between **k_i_** and **k_d_** is much larger in Hg_2_X_2_ than in TeO_2_. Furthermore, the acoustic vector **k_a_** needs to be longer than **k_a_’** to reach the extraordinary wavevector ellipse, which means that diffraction in Hg_2_X_2_ crystals requires a higher driving frequency. Note that for the same crystal, the refractive index values at different wavelengths are not identical, as shown in Table 1. Typically, the refractive indices of ordinary light (o-light) and extraordinary light (e-light) decrease with an increase in wavelength but do not do so in direct proportion; thus, the birefringence of the crystal also varies with different wavelengths. Table 1 lists the refractive indices of acousto-optic crystals at typical visible wavelengths and long-wave infrared wavelengths. The refractive index of TeO_2_ at long-wave infrared wavelengths is not listed because it is not transparent to the long-wave infrared spectrum. As the wavelength changes from 632.8 nm to 10,600 nm, the change in the refractive index of e-light is much more dramatic than that of o-light. The birefringence of a crystal is often described by Δn = |n_o_ − n_e_|. However, Δn is not suitable for representing birefringence in the analysis of separation angles. Assume there are two types of acousto-optic crystals. In their phase-matching condition, the refractive indices of ordinary light are n_o1_ and n_o2_ and those of extraordinary light are n_e1_ and n_e2_, respectively. If n_o1_/n_o2_ = n_e1_/n_e2_, then even if one of the crystals has a larger Δn, it will not result in a larger internal separation angle because the phase-matching triangles in the two crystals are similar.

For a fabricated AOTF, the crystal and the acoustic cut angle are fixed. At this point, the incident angle determines the separation angle inside the crystal. As illustrated in Figure 1c, with a fixed acoustic angle *α*, as the incident angle increases from *θ_i_* to *θ_i_*’, the acoustic wavevector required for phase matching changes from **k_a_** to **k_a_’** and the internal separation angle changes from Δ*θ* to Δ*θ*’. The relationship between the internal separation angle and the incident angle is not always monotonic. When the value of α is small (such as α = 5°), the internal separation angle inside the crystal first increases and then decreases with the increase in the incident angle.

When the incident angle and crystal are fixed, the acoustic cut angle determines the separation angle inside the crystal. As shown in Figure 1d, with the incident-light direction fixed at *θ_i_*, as the acoustic cut angle increases from *α* to *α*’, the required acoustic wavevector for phase matching changes from **k_a_** to **k_a_’** and the internal separation angle inside the crystal changes from Δ*θ* to Δ*θ*’. When the incident light is o-light and the diffracted light is e-light, the internal separation angle is smallest when **k_i_** and **k_a_** are collinear. As **k_a_** starts to rotate from the collinear orientation, the acoustic angle increases and the internal separation angle also increases. When e-light is incident and o-light is diffracted, the change in the internal separation angle with the acoustic angle is more complex. The internal separation angle is smallest when **k_i_** and **k_a_** are collinear. As **k_a_** starts to rotate from the collinear orientation, the internal separation angle continuously increases until the acoustic wavevector **k_a_** rotates to be tangent to the wavevector circle of the o-light.

Define the parameter *δ* = n_o_/n_e_ as the ratio of refractive indices along the crystal’s primary axis. A straightforward conclusion can be drawn from Equation (2): for the same incident wavevector and acoustic cut angle, a larger separation angle can be achieved with crystals of smaller refractive-index ratios *δ* = n_o_/n_e_. In this study, both tellurium dioxide and mercurous bromide/chloride crystals are positive uniaxial crystals with *δ* < 1.

When considering the precise calculation of the separation angle, the refractive index of the e-light should not be approximated by the principal axis refractive index. Instead, it should be calculated based on the direction of the optical wavevector. Therefore, when the incident light is the e-light and the diffracted light is the o-light, the separation angle inside the crystal Δ*θ*_e→o_ satisfies the following equation:

(3) $$\cos(\Delta \theta_{e\to o}+\theta_{i}-\alpha )=\frac{1}{\delta }\cos(\theta_{i}-\alpha )\sqrt{\frac{\delta^{2}}{\delta^{2}\sin^{2}\theta_{i}+\cos^{2}\theta_{i}}}$$

For o-light incidence and e-light diffraction, the separation angle inside the crystal Δ*θ*_o→e_ satisfies the following equation:

(4) $$\cos(\Delta \theta_{o\to e}+\theta_{i}-\alpha )=\delta \cos(\theta_{i}-\alpha )\sqrt{\sin^{2}\theta_{d}+\frac{1}{\delta^{2}}\cos^{2}\theta_{d}}$$

Additionally, due to dispersion, the refractive indices vary with wavelength. As the refractive indices of ordinary and extraordinary light do not vary proportionally, when the incident angle and the acoustic angle are fixed, the ratio *n_i_*/*n_d_* differs for various wavelengths, affecting the achievable separation angles.

**Figure 2:**
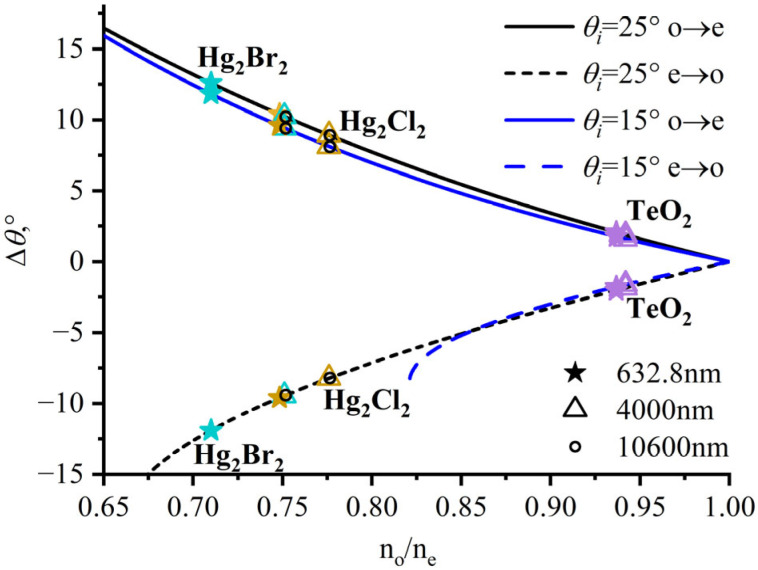
Dependence of separation angle inside the crystal Δ*θ* on the ratio of refractive indices when *α* = 6.5°. Purple represents TeO_2_; yellow represents Hg_2_Cl_2_; and cyan represents Hg_2_Br_2_.

Compared to tellurium dioxide, mercurous bromide/chloride crystals exhibit larger birefringence, with smaller n_o_ and higher n_e_. As plotted in Figure 2, solid lines represent incident light as o-light and diffracted light as e-light; dashed lines represent incident light as e-light and diffracted light as o-light. With different polarization incidents, the diffracted light is distributed on different sides of the incident direction.

For TeO_2_-based AOTF devices, the value of the separation angle is relatively small. Correspondingly, the separation angles under different wavelengths also vary within a small value range. For instance, when the directions of the acoustic wavevector and the incident light vector are fixed (*α* = 6.5° and *θ_i_* = 15°), with variations from visible light to the mid-wave infrared spectrum, the separation angle inside the crystal Δ*θ*_o→e_ ranges from 1.6° to 1.8°, while the separation angle outside the crystal *θ_sep_* ranges from 3.4° to 3.5°. When the directions of the acoustic wavevector and the wavelength of light are fixed (*α* = 6.5° and *λ* = 632.8 nm), as the direction of the incident light vector varies from 15° to 25°, the separation angle Δ*θ*_o→e_ inside the crystal ranges from 1.8° to 2.1°, while the external separation angle *θ_sep_* ranges from 3.5° to 4.1°. The restriction imposed by the small separation angle significantly limits the light flux of spectral imaging systems.

In the development of AOTF devices using mercurous halide crystals, the small refractive index ratio *δ* = n_o_/n_e_ allows for larger separation angles. For example, designs setting *α* = 6.5° and *θ_i_* = 15° or 25° consistently yield a separation angle inside the crystal Δ*θ* above 7°, while the corresponding separation angle outside the crystal *θ_sep_* can reach 10° or even 20°. However, in the visible-to-short-wave infrared spectrum, mercurous halide materials display substantial dispersion, causing significant discrepancies in separation angles given a fixed crystal-cut device at varying wavelengths.

This complexity poses challenges in designing optical systems for imaging across a broad spectral range. Nonetheless, in the mid-to-long-wave infrared spectrum, the refractive indices change little with wavelength, causing the separation angle to remain mostly unchanged as the wavelength varies. As shown in Figure 2, the hollow circles representing the separation angle of the 10,600 nm wavelength overlap with the triangles representing the separation angle of the 4000 nm wavelength, suggesting that the dispersion of Hg_2_X_2_ crystals is not significant in the infrared spectrum and that their birefringence properties are comparatively stable. In contrast, the separation angles at the visible-light wavelengths, denoted by the star symbols, are far from the hollow triangles and circles, indicating that the dispersion-induced changes in separation angles have a notable impact on the visible-to-near-infrared spectrum.

## 3. Results and Discussion

**Figure 3:**
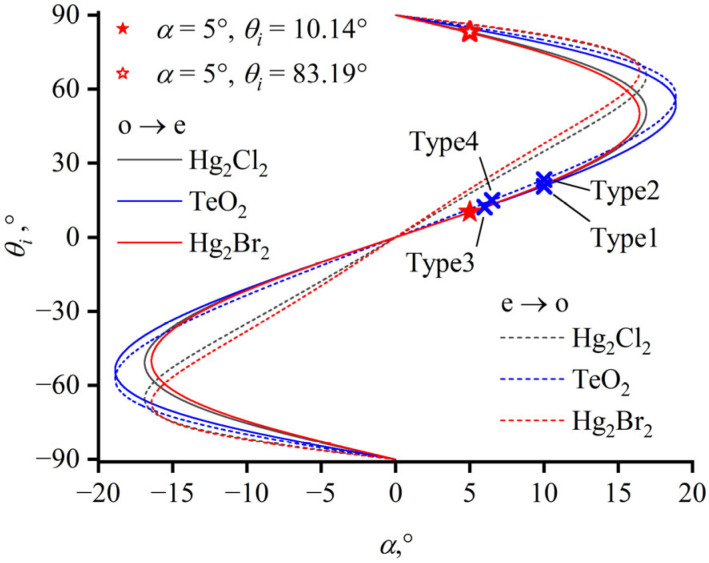
Dependence of incident angle vs. acoustic angle under PTP. The reported configurations are marked. Type 1: *α* = 10°, *θ_i_* = 20.7°, o→e [25]; Type 2: *α* = 10°, *θ_i_* = 23.4°, e→o [27]; Type3: *α* = 6°, *θ_i_* = 12.2°, o→e [26]; Type 4: *α* = 6.5°, *θ_i_* = 15°, e→o.

Presently, most TeO_2_-based AOTF device designs adhere to the PTP [25,26]. This principle dictates that the tangent of the incident ellipsoid at the endpoint of the incident wavevector is parallel to that of the diffracted wavevector. Consequently, the PTP in the design results in a restriction: for a certain acoustic vector direction, only a pair of incident angles can achieve phase matching with a specific wavelength, as depicted in Figure 3. For instance, with a wavelength of 632.8 nm and an acoustic cut angle *α* of 5°, the incident angles *θ_i_* in crystallographic coordinates are 10.14° and 83.19° for Hg_2_Cl_2_ under PTP, as marked with star symbols in Figure 3. The necessary ultrasonic driving frequencies are 55 MHz and 155 MHz, respectively. In practical device design, it is common to adopt smaller incident angles based on the PTP, as marked with a solid star symbol in Figure 3, as designing angles close to 90° for incidence makes the fabrication of devices complicated.

**Figure 4:**
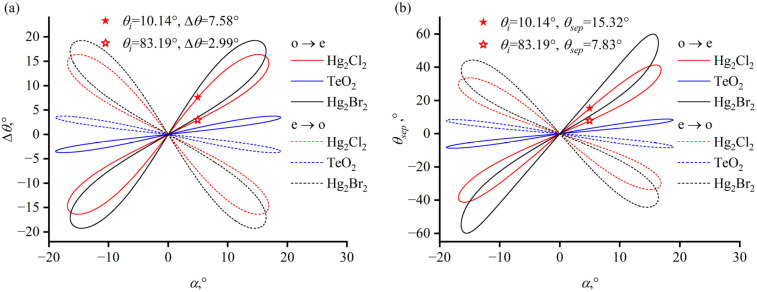
Dependence of separation angle on acoustic angle. (**a**) Dependence of separation angle inside the crystal Δ*θ* vs. acoustic angle *α* under PTP. (**b**) Dependence of separation angle outside the crystal *θ_sep_* vs. acoustic angle *α* under PTP when the incident surface and the exit surface of the crystal are parallel to each other.

There is a maximum value of acoustic cut angle, α_max_, in AOTF design; this value is based on the PTP. When the acoustic cut angle exceeds α_max_, no incident angle satisfies the PTP. For TeO_2_, α_max_ is 18.9°; for Hg_2_Cl_2_, α_max_ is 16.9°; and for Hg_2_Br_2_, α_max_ is 16.4°. Correspondingly, there is also a maximum value for the separation angle inside the crystal. As illustrated in Figure 4a, for the same crystal with the same polarization state of incident light, each α corresponds to two ordinate values, representing two separation angles inside the crystal under the PTP. In Figure 4, the color of each line indicates the type of crystal and the line style indicates the type of incident polarization state. The blue graph representing the separation angle in a TeO_2_ crystal is more flattened, signifying that the separation angle in TeO_2_ is smaller than those represented by the red and black lines (mercurous chloride and bromide crystals, respectively). At acoustic cut angle α = 5°, a larger internal separation angle of 7.58° is obtained when the incident angle is 10.14°, as marked with a solid star symbol in Figure 4, while the internal separation angle for an incident angle of 83.19° is 2.99°, as marked with a hollow star symbol.

The separation angle outside the crystal is also determined by the cut angle of the exit surface. The separation angle outside the crystal, as shown in Figure 4b, is calculated assuming that the exit surface is parallel to the incident surface. The refraction at the exit surface causes the external separation angle to display an asymmetrical characteristic. At acoustic cut angle α = 5°, the separation angle outside the crystal *θ_sep_* is 15.32° for an incident angle of 10.14°, and a separation angle of 7.83° is obtained when the incident angle is 89.19°, as indicated by the star symbols in Figure 4b.

In AOTF design, the cut angle of the exit surface can be designed according to different design requirements, such as compensation for chromatic aberration of diffracted light [28] or multiplexed polarization [29]. It is also possible to further enlarge the separation angle outside the crystal by adjusting the exit surface cut angle. However, it should be noted that due to the larger e-light refractive index of the Hg_2_X_2_ crystal and the larger separation angle inside the crystal, it is easier for the diffracted light to undergo total internal reflection at the exit surface, resulting in the diffracted light being unable to exit. For TeO_2_, the minimum critical angle for total reflection is 24.5°; for Hg_2_Cl_2_, it is 22.4°; and for Hg_2_Br_2_, it is 19.6°.

**Figure 5:**
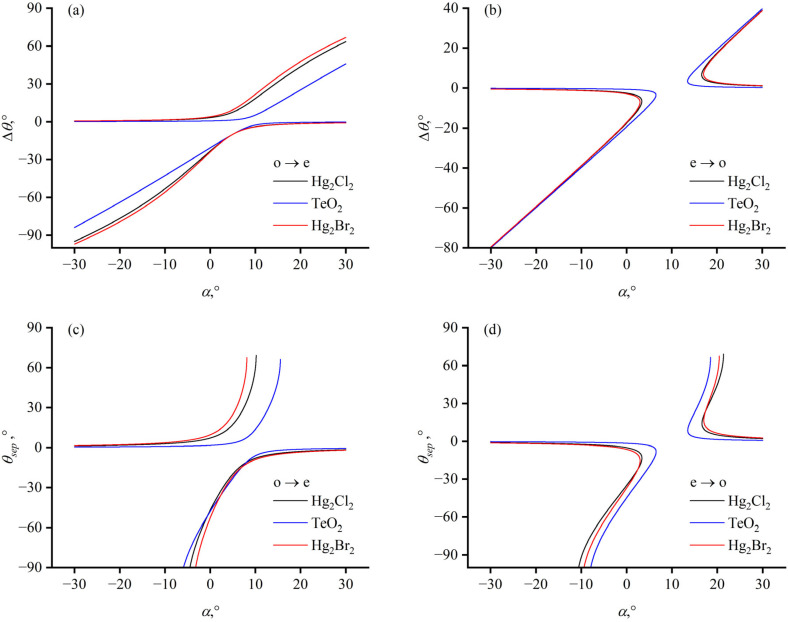
Dependence of separation angle on acoustic angle α with an incident angle of *θ_i_* = 10°. (**a**,**b**) show the dependence of separation angle inside the crystal Δ*θ* on acoustic angle α for ordinary and extraordinary incidence, respectively. (**c**,**d**) show the dependence of separation angle crystal outside the crystal *θ_sep_* on acoustic angle α for ordinary and extraordinary incidence, respectively.

**Figure 6:**
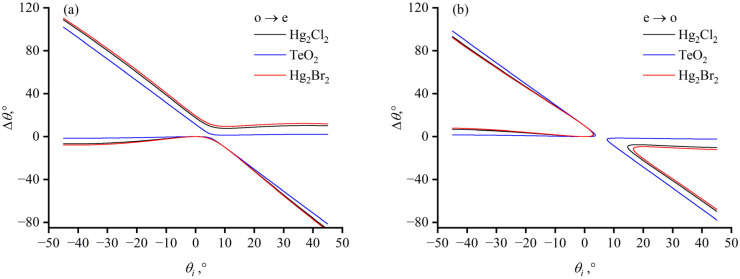

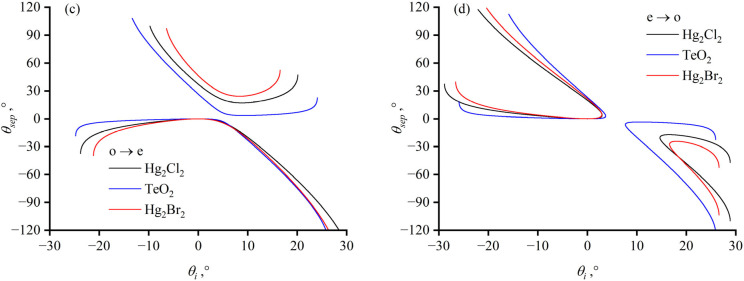
Dependence of separation angle on incident angle *θ_i_* with an acoustic angle of α = 5°. (**a**,**b**) show the dependence of the separation angle inside the crystal Δ*θ* on incident angle *θ_i_* for ordinary and extraordinary incidence, respectively. (**c**,**d**) show the dependence of the separation angle *θ_sep_* outside the crystal on incident angle *θ_i_* for ordinary and extraordinary incidence, respectively.

When we allow the acoustic angle and the incident angle to not satisfy the PTP, it is still possible to achieve phase matching for a specific wavelength by matching the ultrasonic frequency to obtain monochromatic diffracted light. This is the common operational scenario for AOTF devices used in spectral imaging. The PTP is employed in the design phase for a maximum angular aperture at a central wavelength of the AOTF device. Upon completion of the design and fabrication of the devices, to tune the spectral channels by adjusting the driving frequency, wavelengths other than the specially designed central wavelengths all operate with PTP unsatisfied. Furthermore, recent AOTF device designs that consider polarization or chromatic aberrations are also not limited to satisfying the PTP [29]. Hence, we provide the variations in separation angles with the acoustic angle α when the incident angle *θ_i_* = 10° (Figure 5) and the variations in separation angles with the incident angle *θ_i_* when the acoustic angle α = 5° (Figure 6).

When the incident light is o-light, for a positive uniaxial crystal, the o-light circle is inscribed within the e-light ellipse. For any acoustic angle, there always exists a pair of ultrasonic driving frequencies that achieve phase matching, as shown in Figure 5a,c; that is, Equation (4) always has two solutions. However, when the incident light is e-light, there exists a range of acoustic angle values where Equation (3) has no solution and no diffracted light is produced, as depicted in Figure 5b,d. As the incident light wavevector approaches the [001] axis, the non-diffracted region diminishes into the origin and the variation in the separation angle is nearly linear. The external separation angle, *θ_sep_*, as shown in Figure 5c,d, is calculated under the condition that the exit surface normal is along the [001] axis. Due to refraction at the exit surface, the external separation angle is larger than the separation angle inside the crystal and it varies to a greater extent with the angle of the acoustic wave. When the diffracted light arrives at the exit surface at an angle that exceeds the critical angle for total internal reflection, the diffracted light cannot exit.

Similarly, when the incident light is o-light, for any incident angle, there always exist two sets of phase-matching relationships that result in diffraction light wavevectors on both sides of the incident light wavevector, producing a pair of separation angles with different magnitudes and opposite signs, as shown in Figure 6a,c. When the incident light is e-light, there is a certain range of incident angles within which no diffraction light is generated, as illustrated in Figure 6b,d. As the acoustic wave vector approaches the [110] axis, the region of no diffraction for the e-light incident diminishes to the origin, and the change of separation angle is approximately linear when the absolute value of the incident angle is small.

Practical device designs often adopt small acoustic cut angles due to the significant acoustic anisotropy of tellurium dioxide and mercurous halide crystals, leading to pronounced acoustic walk-off phenomena. At α = 5°, the acoustic walk-off angle of a TeO_2_ crystal is 40.8°; that of a Hg_2_Cl_2_ crystal is 35.5°; and that of a Hg_2_Br_2_ crystal is 43.2°.

**Figure 7:**
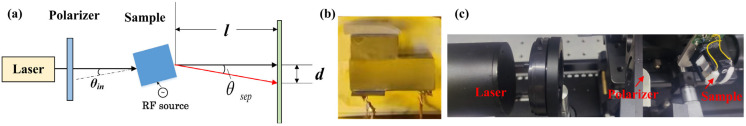
(**a**) Experimental setup for measuring separation angles, (**b**) the photograph of the tested sample, and (**c**) the photograph of the experiment.

Experiments were performed to investigate phase matching in tellurium dioxide and mercurous bromide/chloride crystals using the setup illustrated in Figure 7a. The experimental setup consists of a laser, a polarizer, the sample to be tested, and a light-receiving screen. In the experiment, a He-Ne laser (Daheng Optics, Beijing, China) and a carbon dioxide laser (SPT Laser, Dongguan, China) are used to obtain incident light with wavelengths of 632.8 nm and 10,600 nm, respectively. Polarizers (Daheng Optics, Beijing, China) with different transmission axes are installed in front of the sample to ensure that the light on the incident surface is the ordinary or extraordinary light required in the experiment. The sample to be tested is fixed on a rotating stage to allow the experimenter to change the angle between the incident laser and the crystal incident surface. The laser is incident onto the sample surface at an angle of *θ_in_*. Here, the incident angle *θ_in_* relates to the angle of incidence in the crystallographic coordinate through a conversion relationship sin(*θ_in_*) = *n_i_*sin(*θ_i_* − *θ_c_*). The ultrasonic driving frequency applied to the transducer was tuned, allowing the condition at which the diffracted light reached its maximum intensity to be identified as the phase-matching condition. Samples with two crystals cut along the crystallographic axis and utilizing the same ultrasonic transducer were fabricated, as shown in Figure 7b. As a result of using the same transducer, the acoustic waves in both crystals have the same acoustic angle α. By measuring the distance *d* between the transmitted light and the diffracted light on the screen as well as the distance *l* from the exit surface of the sample to the light screen, the external separation angle *θ_sep_* can be obtained through the following relationship: *θ_sep_* = arctan(*d*/*l*).

**Figure 8:**
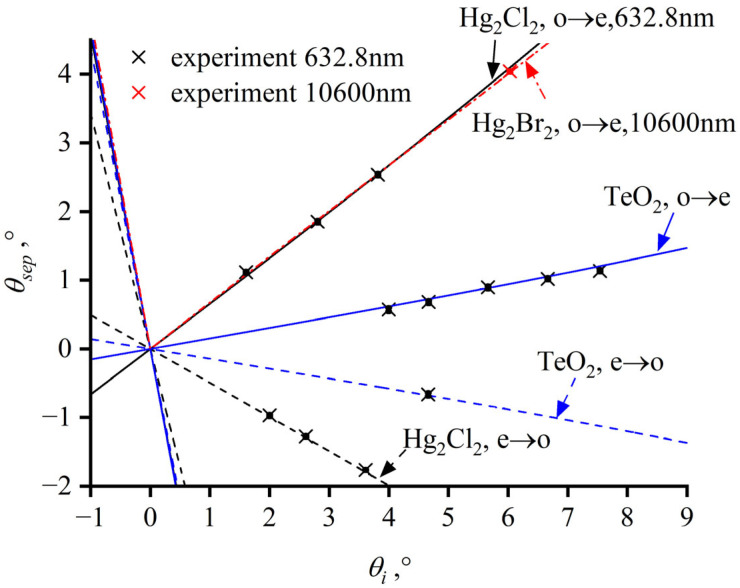
Theoretical curves of the separation angles for tellurium dioxide and mercurous halide crystals with an acoustic angle cut of 0° as a function of the incident angle. The blue line represents TeO_2_; the black represents Hg_2_Cl_2_; and the red represents Hg_2_Br_2_; dashed lines and solid lines indicate the incidence of e-light and o-light, respectively, at a wavelength of 632.8 nm, and dot-dashed lines represent the incidence of o-light in Hg_2_Br_2_ at a wavelength of 10,600 nm.

Laser beams of varying incident angles *θ_in_* enter the crystal with different polarizations at a wavelength of 632.8 nm in the experiment setup shown in Figure 7c. The incident angle is adjusted by rotating the crystal. For TeO_2_, the o-light incident angles range from 4–7.5° and the experimental results are consistent with the theoretical curve represented by the blue solid line. For Hg_2_Cl_2_, the o-light incident angles range from 1.6–4° and e-light incident angles range from 1.9–3.6°. The measurement results align with the theoretical separation angles for o-light, indicated by the black solid line, and those for e-light, indicated by the black dashed line (Figure 8). The separation angle for Hg_2_Br_2_ is measured at a wavelength of 10,600 nm, with an incident angle of 6°. As shown in Figure 8, the measured separation angle of 4.04°, marked with a red cross symbol, corresponds well the theoretical values, which are indicated by the red dash-dot-line. With the acoustic angle of zero and incident angles *θ_in_* less than 10°, the separation angle *θ_sep_* is nearly linear in relation to the incident angle *θ_in_*. However, as the absolute value of the incident angle increases, the slope of the separation angle also increases.

In recent years, other studies on the geometry of acousto-optic interactions and the design of mercuric halides have been conducted. The excellent properties of novae crystals and PTP have been utilized to achieve a large monochromatic angular aperture and high spectral resolution in the long-wave infrared spectrum [11]. By analyzing the acousto-optic phase-matching geometry, multiple acousto-optic interaction relations with the same acoustic angle that satisfy PTP within the same crystal have been considered to increase the optical throughput [12]. Different crystals have been used under PTP to obtain a large monochromatic angular aperture, and the significant role of crystal birefringence has been highlighted [19]. In the common optical schemes for acousto-optic spectral systems summarized in prior work [30], the AOTF is the key factor that determines the overall light throughput of the system. In contrast to these studies, this article focuses on the angular-spectral characteristics of AOTFs that can be used to enhance the system’s optical throughput. We provide a comprehensive theoretical simulation of the separation angle under different parameters while satisfying and not satisfying PTP. The accuracy of the analysis results and that of the simulation model were verified by testing samples of different crystals with the same acoustic angle.

## 4. Conclusions

In this investigation, we explored the angular-spectral properties of diffracted light in non-collinear acousto-optic interactions involving mercurous halide and tellurium dioxide crystals. The separation angle between diffracted and transmitted light is determined by the direction of the incident wavevector, the acoustic wavevector, and the optical anisotropy of the crystal. Increasing the angle between the acoustic wave and the [110] crystal axis results in an enlarged separation angle. Depending on the specific application requirements, variations in cutting parameters need to be considered in terms of their impact on separation angle, spectral resolution, diffraction efficiency, and other performance metrics, as modifying the ultrasound cut angle may lead to losses in other aspects. High birefringence can generate large separation angles in acousto-optic diffraction. High birefringence also increases the driving frequency and enhances spectral resolution. Therefore, using high birefringent acousto-optic crystals is cost-effective and minimizes performance losses when the aim is to improve the separation angle of acousto-optic devices. During the AOTF design phase, the methods outlined in this study can aid in selecting the optimal crystal and crystal-cut layouts for specific applications, contributing significantly to the development of high-throughput AOTF spectral imaging systems.

## Figures and Tables

**Table 1:** Properties of acousto-optic crystals [21,22,24].

AO Crystals	Acoustic Velocity (km/s)	n_o_ (632.8 nm)	n_e_ (632.8 nm)	n_o_ (10,600 nm)	n_e_ (10,600 nm)
TeO_2_	Shear [110]0.616, Shear [001]2.104	2.26	2.41	-	-
Hg_2_Cl_2_	Shear [110]0.347, Shear [001]1.084	1.96	2.62	1.90	2.45
Hg_2_Br_2_	Shear [110]0.282, Shear [001]1.008	2.12	2.98	2.03	2.70

## Data Availability

Data are contained within the article.
